# Acarbose for Postprandial Hypotension With Glucose Metabolism Disorders: A Systematic Review and Meta-Analysis

**DOI:** 10.3389/fcvm.2021.663635

**Published:** 2021-05-20

**Authors:** Biqing Wang, Junnan Zhao, Qiuxiao Zhan, Rongyanqi Wang, Birong Liu, Yan Zhou, Fengqin Xu

**Affiliations:** ^1^Graduate School, Beijing University of Chinese Medicine, Beijing, China; ^2^Xiyuan Hospital, China Academy of Chinese Medical Sciences, Beijing, China; ^3^Institute of Geriatrics, Xiyuan Hospital, China Academy of Chinese Medical Sciences, Beijing, China

**Keywords:** acarbose, postprandial hypotension, glucose metabolism disorders, systematic review, meta-analysis

## Abstract

**Background:** Postprandial hypotension (PPH) is an independent predictive factor of all-cause mortality in older people. Drug management has not achieved a satisfactory effect yet. In recent years, many studies have found that acarbose may be effective in the treatment of PPH with glucose metabolism disorders.

**Objective:** To assess the efficacy and safety of acarbose on PPH with glucose metabolism disorders.

**Methods:** PubMed (MEDLINE), Cochrane, EMBASE, Web of Science, Clinical Trials, and relevant Chinese databases were searched from inception to October 1, 2020. Randomized controlled studies of acarbose in the treatment of PPH with glucose metabolism disorders were included. Review Manager 5.3 software was used for quality evaluation and meta-analysis. GRADEpro GDT software was used to GRADE the evidence for the research objectives.

**Results:** A total of 4 randomized controlled studies including 202 participants were identified after screening. The meta-analysis showed that acarbose significantly attenuated the decrease in postprandial systolic blood pressure [weighted mean difference (MD): −9.84, 95% CI: −13.34 to −6.33], diastolic blood pressure (MD: −6.86, 95% CI: −12.89 to −0.83), and mean arterial pressure (MD: −8.10, 95% CI: −12.40 to −3.49) compared with the control group. One study reported a case of adverse reactions that included mild abdominal distension in the acarbose group (4.8%, 1/21). No adverse reactions were reported in the other three studies.

**Conclusion:** Acarbose may attenuate the decrease in postprandial blood pressure and avoid the occurrence of PPH in patients with PPH and abnormal glucose metabolism disorders. More clinical trials are needed to make a clear conclusion.

**Registration:** PROSPERO CRD42020171335.

## Introduction

With the growth of the global aging population, the treatment and prevention of high incidence diseases in the elderly have become a major clinical problem. Postprandial hypotension (PPH) is commonly recognized in the senile and frail elderly. PPH not only occurs frequently in hospitalized or critically ill elderly patients but also has a high incidence rate in the healthy elderly population (1–3). PPH was first reported in 1977, which was a common but less concerning disease (4). Further research found that there was a dose–response relationship between the decrease of postprandial systolic blood pressure (SBP) and mortality (5). It is one of the effective predictors of adverse events, such as falls, syncope, stroke, and coronary events. The latest research has also found that PPH can predict the occurrence of new cardiovascular diseases in patients (3).

The pathogenesis of PPH has not been fully understood. Visceral blood increases after eating in healthy people, which reduces the blood volume of cardiac regurgitation. The activity of the sympathetic nervous system, the postprandial heart rate, and the cardiac output increases. It can compensate for the visceral blood pool and maintain the stability of blood pressure (6). Changes associated with aging and disease in the elderly may decrease sympathetic activation (7), decrease baroreflex sensitivity (8), increase mesenteric artery blood flow (9), and increase the gastric emptying rate and gastrointestinal hormone release (10, 11). The comprehensive effect of multiple etiological factors can weaken the compensatory effect of the cardiovascular system, so normal blood pressure cannot be maintained, resulting in PPH. As the pathogenesis of PPH is complex, the effects of different drug treatments on the adaptive population are different, and drug management has not achieved a satisfactory effect yet (12).

PPH is widespread in patients with glucose metabolism disorders. The incidence of PPH in elderly diabetes inpatients can reach 76.9%, which may be related to long-term unsatisfactory blood glucose control leading to autonomic nervous dysfunction and increasing the susceptibility of PPH (13). In recent years, the efficacy of α-glucosidase inhibitors for PPH combined with glucose metabolism disorders has garnered increased attention. α-glucosidase inhibitors may reduce the fluctuation of postprandial blood pressure by inhibiting the absorption of carbohydrates in the brush border of the small intestine and slowing down the gastric emptying rate (12). Meanwhile, it can inhibit the rise of postprandial blood glucose and maintain the blood glucose level.

Acarbose, an α-glucosidase inhibitor, can competitively inhibit the sucrase and glucoamylase at the brush border of villous mucosal cells of the small intestine and delay the digestion and absorption of sucrose and starch. Thus, it is better suited for Asian people who mostly eat carbohydrates (14), with better pharmacokinetic properties, less oral absorption, and lower toxicity. It may be the best drug for the combination treatment of PPH with glucose metabolism disorders (15). At present, many studies have found that acarbose can decrease the range of postprandial blood pressure drop, shorten the duration of hypotension, and reduce blood pressure fluctuation (13, 16–20). However, the clinical research studies generally include small sample size tests. The comprehensive analysis and systematic evaluation of the treatment are lacking.

In this study, we conducted a systematic review and meta-analysis of the data from clinical trials to evaluate whether acarbose is a considerable and safe treatment approach for PPH with glucose metabolism disorders.

## Methods

### Protocol and Registration

The study was based on the Preferred Reporting Items for Systematic Reviews and Meta-Analysis (PRISMA) statement. The systematic review and meta-analysis protocol were registered at the International Prospective Register of Systematic Reviews (PROSPERO).

### Inclusion Criteria

- The included participants were adults aged 18 or older, who met the diagnostic criteria for PPH and glucose metabolism disorders. Diagnostic criteria for PPH: there is still a lack of standardized diagnostic criteria. It is generally recognized that the fall of postprandial SBP of 2 h after meals ≥20 mmHg (1 mmHg = 0.133 kPa) or preprandial SBP ≥100 mmHg and postprandial SBP <90 mmHg, if the maximum decrease range of SBP at 2 h after meals is not up to the above standard, but clinical symptoms have already appeared, such as dizziness and syncope. It also can be diagnosed as PPH (6). Diagnosis standard of glucose metabolism disorders: according to the WHO classification in 1998 (21), glucose metabolism disorders include impaired fasting glucose, impaired glucose tolerance (IGT), and diabetes mellitus (DM).
- Acarbose was used as an intervention drug (dosage form and manufacturer were not limited), whereas placebo or no special intervention was used as a comparison intervention.
- Fluctuations in blood pressure within 2 h after meals including SBP, DBP (diastolic blood pressure) and MBP (mean arterial blood pressure) and the effective rate of drug intervention were considered as the primary outcome indicators. The secondary outcome indicators could include the adverse reactions, symptoms, blood glucose changes, etc.
- The duration of intervention was limited to breakfast time in this study. Previous studies have shown that breakfast time has the most significant drop in blood pressure after meals and is more prone to adverse events (22).

### Exclusion Criteria

- Not meeting the inclusion criteria or incomplete clinical trials.
- Reviews and systematic reviews, basic studies, animal studies, research protocols, and letters.

### Search Strategy

To improve the recall ratio, English databases used “acarbose,” “postprandial hypotension,” “postprandial period,” “PPH,” and “hypotension” as the subject words, combined with their entry terms and abbreviated terms to search PubMed (MEDLINE), Cochrane, EMBASE, Web of Science, and Clinical Trials. Chinese databases used the keywords of “acarbose” and its synonyms and “postprandial hypotension” and its synonyms to search the CNKI, WanFang, CBM, and VIP databases. We also searched conference papers, degree papers, and other gray literature. Manual retrieval included the references of published literature and professional studies. The language was limited to Chinese and English. The starting and ending times of the retrieval were from the establishment of the literature database to October 1, 2020. The detailed database retrieval strategy takes the EMBASE database as an example (Supplementary Table 1).

### Studies Selection and Quality Evaluation

Two researchers (BW and JZ) independently screened the found studies according to the inclusion and exclusion criteria. Inconsistent or uncertain opinions of the included studies were judged by a third person (QZ).

Two researchers (RW and BL) independently evaluated the included literature item by item, and different results were determined by a third person (YZ) if necessary. The “risk of bias” according to the Cochrane Handbook was used for the quality evaluation of the included literature. The evaluation grade included three levels of “low risk, unclear, and high risk,” and finally the risk assessment chart of bias was formed.

Two researchers (BW and YZ) evaluated the reliability of each primary outcome using GRADEpro GDT online software according to the GRADE standard, including overall study design, risk of bias, inconsistency, imprecision, and other considerations. Any inconsistencies in the assessment results were resolved by negotiation.

### Statistical Analysis of Literature Data

The data of the included literature were extracted by two researchers (BW and QZ) independently. Review manager 5.3 was used for data statistical analysis. *Q* test and *I*^2^ test were used to evaluate the heterogeneity.

Outcomes were analyzed using the random effects model to obtain more conservative results as we included different types of abnormal glucose metabolism (DM and IGT) and no restrictions on the dosage forms and manufacturers of acarbose, and heterogeneity was inevitable.

The continuous variable selected weighted mean difference (MD) or standard mean difference (SMD) as the effective index, and the odds ratio (OR) of the two categorical variables was selected as the effective index. If necessary, subgroup analysis was conducted to analyze the causes of heterogeneity. The sensitivity was realized by excluding a single study in turn and then analyzing the remaining studies. If 10 or more trials were included, publication bias would be evaluated by funnel plot.

## Results

### Included Studies Characteristics

**Figure 1:**
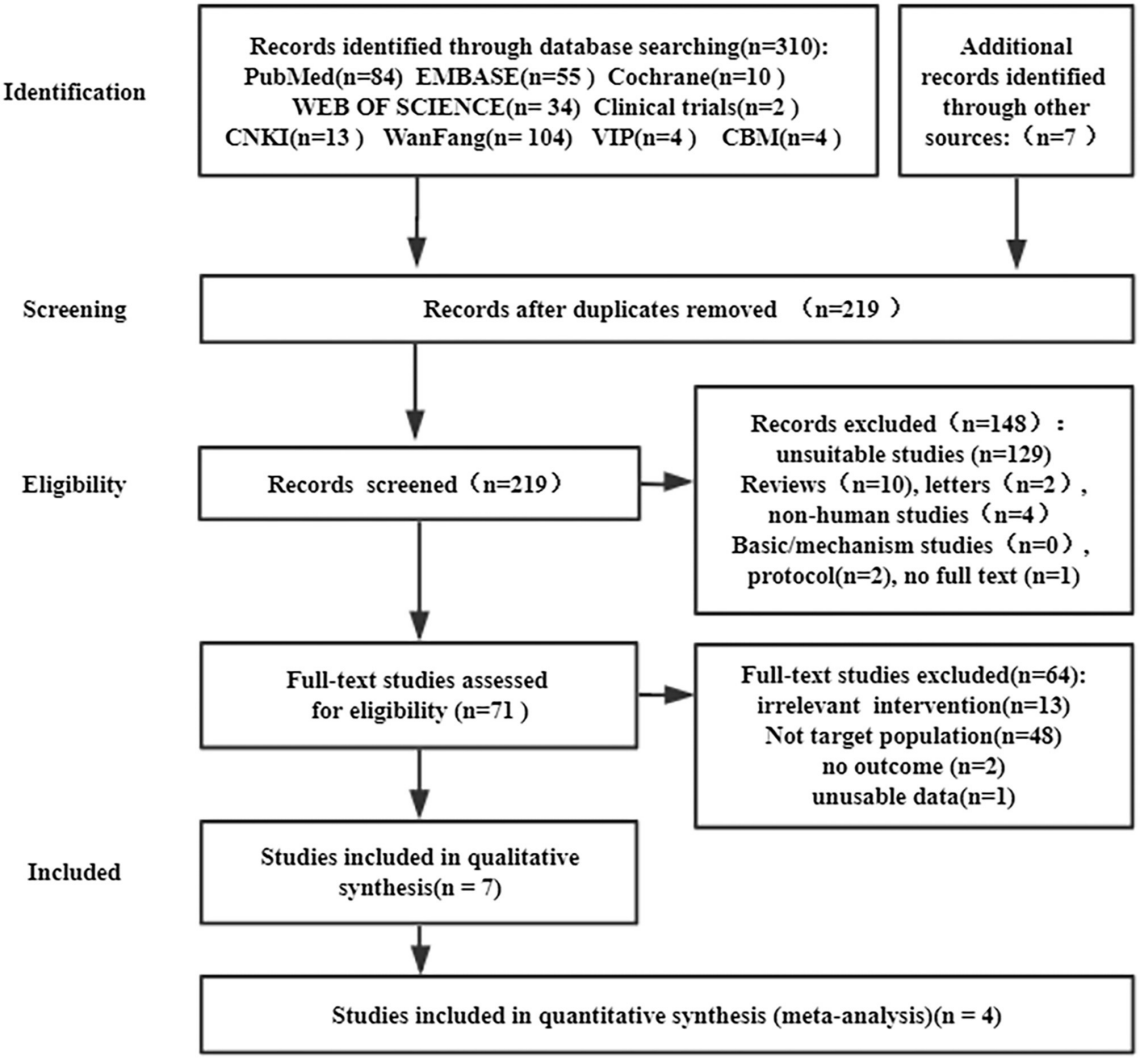
PRISMA flow diagram of the literature search process.

In total, 317 studies were retrieved, including 310 studies retrieved by databases and 7 studies retrieved by manual retrieval. In the end, four randomized controlled studies were included in the meta-analysis (Figure 1).

The included studies met the inclusion criteria, involving 202 patients. These studies were published in recent years (2014–2019). The age of all the participants was ≥60 years old. These studies used acarbose as an intervention with 50 or 100 mg each time, once daily at breakfast time, monitored changes in blood pressure 2 h after breakfast. The characteristics and results of the included studies are summarized in Table 1.

**Table 1:** Characteristics of the included studies.

**References**	**Study design**	**Sample size (N_**I**_/N_**C**_)**	**Mean age (range)**	**Patients**	**Intervention (N_**I**_/N_**C**_)**	**Pre-treatment**	**Testing time**	**Primary outcomes (maximum blood pressure drop)**	**Other outcomes**	**Treatment course**	**Adverse reaction**
Zhang (13)	RCT	20/20	66.68 ± 6.73	PPH + T2DM	Acarbose 100 mg qd/placebo	Fasting 10 h	Breakfast time	SBP: N_I_ < N_C_ (*P* < 0.05) DBP: N_I_ < N_C_ (*P* < 0.05) MBP: N_I_ < N_C_ (*P* < 0.01)	Duration of hypotension: N_I_ < N_C_ (*P* < 0.05) BPV: N_I_ < N_C_ (*P* < 0.05) HR: *P* < 0.05	NS	NS
Zhang et al. (16)	RCT	31/30	60–80	PPH + T2DM	Acarbose 100 mg qd/placebo	Overnight fasting	Breakfast time	SBP: N_I_ < N_C_ (*P* < 0.001) DBP: N_I_ < N_C_ (*P* = 0.004) MBP: N_I_ < N_C_ (*P* < 0.001)	Duration of hypotension: NI < NC (*P* < 0.001) BPV: NI < N (*P* ≤ 0.033) HR: *P* > 0.05	NS	NS
Qiao et al. (17)	RCT	21/22	81–95	PPH + IGT/T2DM	Acarbose 50 mg qd/placebo	Fasting 12 h	Breakfast time	SBP: N_I_ < N_C_ (*P* < 0.05) DBP: *P* > 0.05 MBP: NS	SMABF: N_I_ < N_C_ (*P* < 0.05) PBG: N_I_ < N_C_ (*P* < 0.05)	2 weeks	N_I_: mild abdominal distension (one case, 4.8%) N_C_: none
Peng et al. (18)	RCT	30/28	62–95	PPH + T2DM	Acarbose (NS)/placebo	Fasting 12 h	Breakfast time	SBP: N_I_ < N_C_ (*P* < 0.05) MBP: N_I_ < N_C_ (*P* < 0.05) DBP: N_I_ < N_C_ (*P* < 0.05)	NS	1 week	NS

*RCT, randomized controlled trial; qd, once a day; N_I_, intervention group; N_C_, control group; PPH, postprandial hypotension; T2DM, type 2 diabetes mellitus; SBP, systolic blood pressure; DBP, diastolic blood pressure; MBP, mean arterial blood pressure; P <0.05, have statistical difference; P > 0.05, no statistical difference; BPV, blood pressure variability; NS, not specified; IGT, impaired glucose tolerance; SMABF, superior mesenteric artery blood flow; PBG, postprandial blood glucose*.

### Included Studies Quality Evaluation

**Figure 2:**
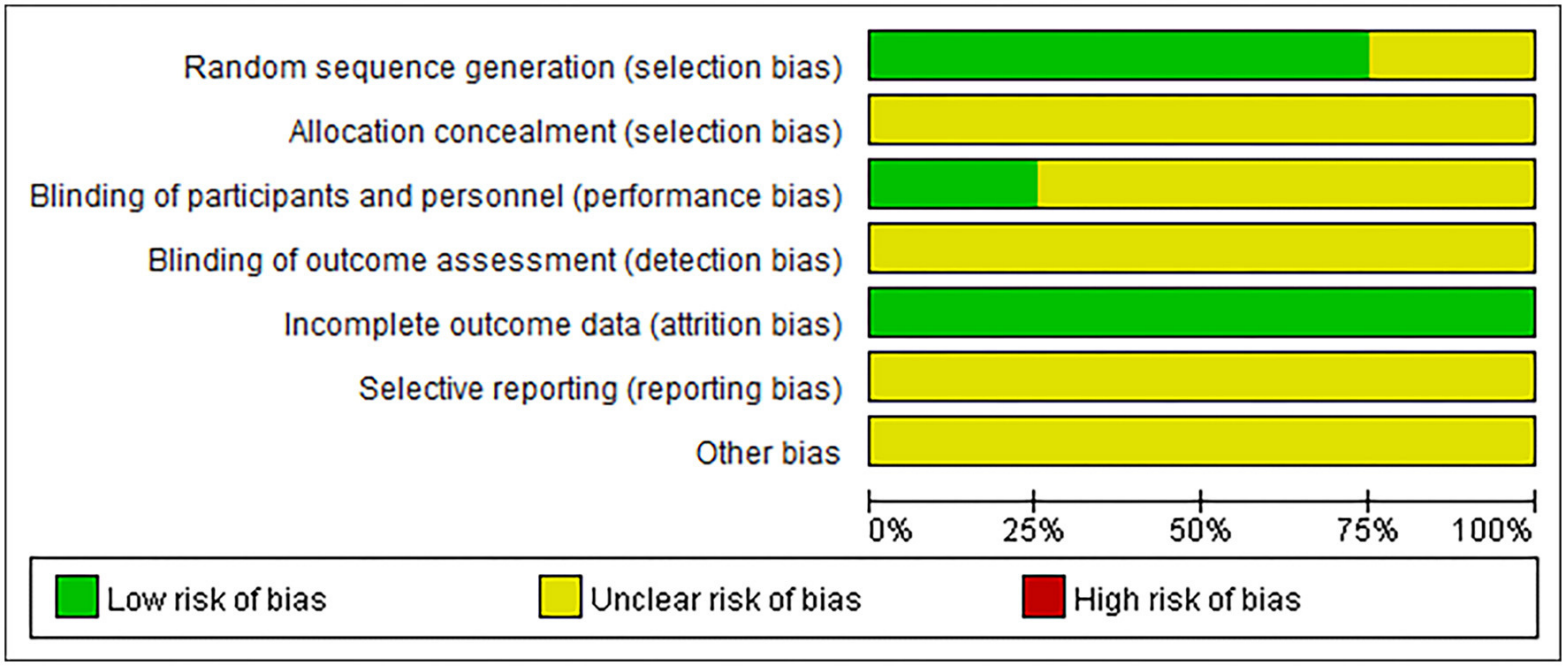
Risk assessment of bias.

Four randomized controlled trials were evaluated according to the risk of bias from the Cochrane Handbook. All the included trials were evaluated as having a certain bias risk (Figure 2). Three of the trials reported using the specific random method (13, 16, 17). All studies did not mention the allocation concealment method. One study reported the single-blind method for patients (16). All the studies did not have sufficient reports to determine the bias of selective reporting results. Other biases were not clear and lacked evaluation criteria.

### Results of Postprandial Blood Pressure

**Figure 3:**
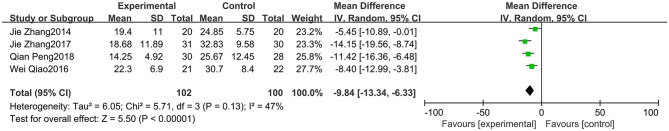
Forest plot of decrease of systolic blood pressure (SBP).

The meta-analysis of postprandial SBP: The results of the meta-analysis showed that acarbose could significantly attenuate the decrease of postprandial SBP compared with placebo (MD: −9.84, 95% CI: −13.34 to −6.33) (Figure 3). All the four included studies reported the maximum fluctuation of SBP at 2 h after the breakfast meal, involving 202 patients. The heterogeneity test showed low heterogeneity (*P* = 0.13, *I*^2^ = 47%).

**Figure 4:**
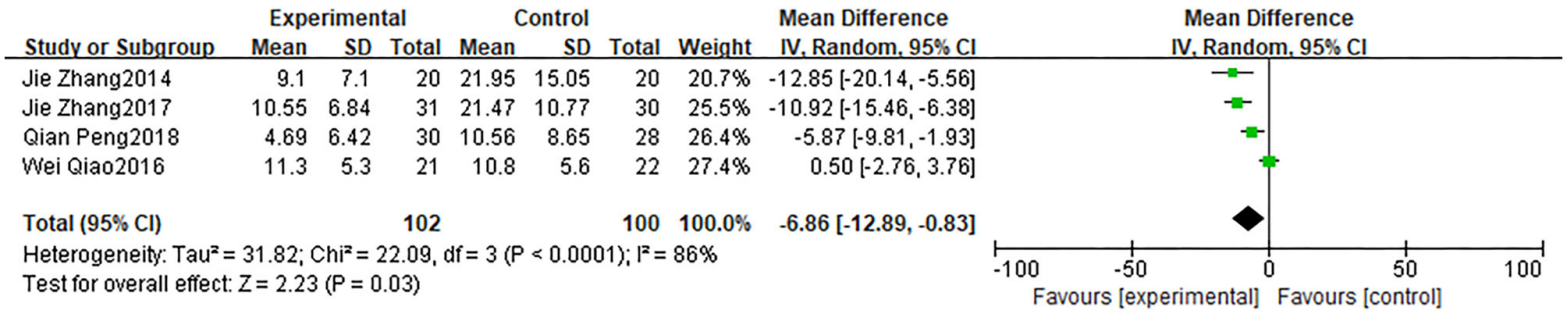
Forest plot of decrease of diastolic blood pressure (DBP).

**Figure 5:**
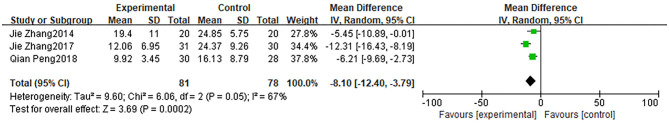
Forest plot of decrease of mean arterial blood pressure (MBP).

The meta-analysis of postprandial DBP and MBP: The results showed that acarbose could attenuate the decrease of DBP (Figure 4) (MD:−6.86, 95% CI: −12.89 to −0.83) and MBP (Figure 5) (MD: −8.10, 95% CI: −12.40 to −3.79). Four studies reported maximum fluctuation of DBP at 2 h after meals, three studies reported that acarbose intervention could better maintain postprandial DBP, and one study reported no statistical difference (17). Three studies reported that acarbose intervention could better maintain postprandial MBP. However, the heterogeneity of the two groups was higher (DBP: *P* < 0.01, *I*^2^ = 86%; MBP: *P* = 0.05, *I*^2^ = 67%), which affected the reliability of the conclusion.

### Sensitivity Analysis and Publication Bias

Sensitivity analysis by eliminating single studies at a time confirmed the stability of SBP and MBP results, but there were some changes in the results of DBP (Supplementary Table 2). DBP changes between the two groups may not be statistically significant. Thus, it is necessary to carefully evaluate the conclusions. Besides, due to <10 articles included, the evaluation of publication bias was not carried out.

### Subgroup Analysis

According to the Cochrane Handbook for Systematic Reviews of Interventions, it is very unlikely that the subgroup analysis will produce useful findings unless there are at least 10 studies in a meta-analysis. The number of studies included in this meta-analysis is <10. Subgroup analysis is not conducive to the correct evaluation of conclusions. Thus, subgroup analysis was not considered.

### Grading of Evidence Quality

According to GRADEpro GDT, the evidence quality was further evaluated, and the GRADE evidence profile was formed (Table 2). The overall quality of evidence for SBP and MBP was low, which was related to the risk of bias and the small sample size. The overall quality of DBP evidence was very low, which is related to the fact that point estimate differences further reduce the level of evidence.

**Table 2:** The GRADE evidence profile for acarbose in the treatment of postprandial hypotension with abnormal glucose metabolism.

**Certainty assessment**							**No. of patients**		**Effect**		**Certainty**	**Importance**
**No. of studies**	**Study design**	**Risk of bias**	**Inconsistency**	**Indirectness**	**Imprecision**	**Other considerations**	**Acarbose**	**Placebo**	**Relative (95% CI)**	**Absolute (95% CI)**		
**SBP**												
4	Randomized trials	Serious^a^	Not serious	Not serious	Serious^c^	None	102	100	–	MD 9.84 lower (13.34 lower to 6.33 lower)	⊕⊕○○ Low	Important
**DBP**												
4	Randomized trials	Serious^a^	Serious^b^	Not serious	Serious^c^	None	102	100	–	MD 6.86 lower (12.89 lower to 0.83 lower)	⊕○○○ Very low	Important
**MBP**												
3	Randomized trials	Serious^a^	Not serious	Not serious	Serious^c^	None	81	78	–	MD 8.1 lower (12.4 lower to 3.79 lower)	⊕⊕○○ Low	Important

*CI, confidence interval; MD, mean difference*.

a *All of the studies mentioned random methods. Three of the studies reported the specific random method. All studies did not mention the allocation concealment method. One study reported the single-blind method for patients*.

b *Wide variance of point estimates*.

c *Small sample sizes and not meet the optimal information size*.

## Discussion

PPH is the height of the severity of diabetes, especially with diabetic autonomic neuropathy (16) and rapid gastric emptying (10). Acarbose is effective in the treatment of abnormal glucose metabolism. Recent studies have shown that acarbose may help improve PPH, so the clinical studies of acarbose in the treatment of PPH with glucose metabolism disorders have attracted more and more attention in recent years (15). Possible mechanisms of acarbose to improve PPH include inhibition of postprandial splanchnic perfusion, which reduces high-risk disease in the elderly, which is related to age. The prevalence of PPH in elderly inpatients can reach 46% (1, 23). The clinical non-drug management of PPH mainly includes eating more meals a day but less food at each, drinking water before meals, postprandial exercise, etc. (24). Currently, there is no recognized drug treatment. PPH is correlated with circulatory volume loss (17) and inhibition of small intestine carbohydrate absorption, slowing gastric emptiness (13). More mechanisms need to be further studied.

In this study, we carried out the meta-analysis of the randomized controlled trials of acarbose for the treatment of PPH with glucose metabolism disorders. To improve the recall rate as much as possible, we searched nine databases and expanded the search scope by perfecting the search formula. Finally, 317 pieces of literature were retrieved, and a total of 4 randomized controlled studies were included. The primary outcomes of the meta-analysis were the changes in blood pressure after meals in the acarbose and placebo groups. The results of the meta-analysis indicate that acarbose is effective in attenuating postprandial blood pressure decrease. Sensitivity analysis showed no significant difference between the results of SBP and MBP, and the overall quality of evidence was low, representing limited confidence in the effect estimate of SBP and MBP. However, the results of DBP were changed by sensitivity analysis, and the overall quality of evidence was very low, representing very little confidence in the effect estimate of DBP. The above results show that acarbose may attenuate the decrease in SBP and mean arterial pressure. More clinical trials are needed to make a clear conclusion.

This study preliminarily proved that acarbose may attenuate the decrease in postprandial blood pressure of patients with PPH and glucose metabolism disorders. Besides, acarbose may reduce postprandial blood pressure fluctuation, shorten the duration of PPH (13, 16), and improve symptoms (25, 26). However, there are few randomized controlled trials of related outcome indicators, which adversely impact the analysis of the reliability of the results through meta-analysis. More studies in the future will provide the possibility for the analysis of the above outcome indicators. To reduce the influence of confounding factors on outcomes, the inclusion criteria for our study protocol required that the measurement time should be breakfast time. However, research methods for PPH should be further standardized in the future, including dose of the medicine, mealtime, reasonable dietary composition, and measurement of body position. It will help to further increase the reliability of outcome indicators and reduce the influence of confounding factors. Currently, highly recommended research methods include: first, patients should be given a standardized mixed diet with liquid food, to facilitate different types of patients to eat at the same time. Second, patients should be asked to fast overnight and measured in the morning, when blood pressure drops the most. Third, the measurement position of patients should be in the sitting or lying position. The sitting position can simulate the position commonly used in real eating, but the sitting position may promote the reduction of blood pressure. Fourth, researchers should avoid the combination of antihypertensive drugs or diuretics in patients, which may aggravate the decrease in blood pressure after meals (27, 28).

Current clinical studies have shown that acarbose treatment has not found serious hypoglycemia and gastrointestinal reactions. Three studies reported adverse reactions, and one randomized controlled study (18) reported that one case of mild abdominal distension (4.8%, 1/22) occurred in the acarbose group. One self-control study reported five cases of mild gastrointestinal reactions (8.3%, 5/60), including three cases of abdominal distension and two cases of diarrhea. The adverse reaction improved immediately after stopping the drug, and no patient had heart and cerebrum ischemic events (29). A case report reported a patient with mild abdominal distention and good tolerance (25). No serious adverse reactions were reported in all studies. Bloating is the most common digestive system adverse reaction of acarbose, but previous studies have shown that acarbose can easily lead to hyperglycemia and hypoglycemia when used together with sulfonylureas, metformin, or insulin. At present, there is a lack of studies on adverse reactions of combined drugs. Therefore, more attention should be paid to the safety issues in clinical use, especially the studies of drug interactions (30).

Besides, the current study subjects were mainly asymptomatic elderly people, and the changes in blood pressure after meals were taken as the main outcome of the study. There were few reports on patients with PPH accompanied by clinical symptoms. Only two cases were reported to improve PPH symptoms in the present studies (25, 26). Improvement of symptoms should be considered as one of the efficacy outcome indicators in the future. Furthermore, follow-up for a certain period should be conducted in future studies to better evaluate the impact on the incidence of adverse events and drug interactions.

Diabetes and PPH have similar pathological changes, including autonomic neuropathy and slow gastric emptying. In addition to acarbose, other hypoglycemic medications have shown potential in the treatment of PPH including glucagon-like peptide-1 (GLP-1) receptor agonist, dipeptidyl peptidase-4 (DPP-IV) inhibitors, and metformin. GLP-1 receptor agonist in patients with type 2 diabetes has the effect of delaying gastric emptying, reducing the increase of superior mesenteric artery blood flow, and attenuating the decrease in postprandial blood pressure. Thus, it may have a certain potential effect for the treatment of PPH (31–33). However, there is a lack of clinical trials on GLP-1 receptor agonists in patients with PPH. In two case reports, DPP-IV inhibitors were found to improve symptoms of dizziness after a meal and decrease blood pressure drop (34, 35), but DPP-IV inhibitors showed no difference in gastric emptying and postprandial blood pressure compared with placebo in the new randomized crossover trial (36), so further research is necessary to confirm the mechanism of the DPP-IV curative effect. Duodenal administration of 1 g of metformin improved the frequency and degree of PPH and delayed gastric emptying in type 2 diabetic patients who were not receiving hypoglycemic medication (37).

The clinical studies of the above three kinds of hypoglycemic drugs had a small sample size, and the curative effect and mechanism were not clear. In addition to the drug treatment, foods with a low glycemic index can slow down gastric emptying and the rate of sugar absorption in the intestine and have a similar mechanism of action to oral hypoglycemic drugs, such as acarbose. Existing studies have shown that patients with PPH who chose the low-carbohydrate diet had the smallest drop in postprandial blood pressure and the shortest duration compared with the high-carbohydrate diet (38). Therefore, further studies should be conducted to confirm the effects of hypoglycemic drugs based on acarbose and the low glycemic index diet on postprandial blood pressure. It will help to further clarify the pathological and physiological relationship between glucose metabolism disorders and PPH and promote the development of prevention and treatment of related diseases.

## Limitations

There are some limitations to the conclusions of this meta-analysis. First, the literature retrieval languages were Chinese and English, and there may exist language bias. Second, although the retrieval scope has been expanded to ensure the recall ratio, the number of studies and sample size meeting the inclusion criteria were still small. Large-scale randomized controlled trials are lacking, which has adverse effects on the reliability of the results and evaluation of publication bias. Third, due to the limitation of the number of included studies, it was not appropriate to apply subgroup analysis and meta-regression analysis to explain the source of heterogeneity and lack of analysis of heterogeneity and treatment of confounding factors. Finally, the research design of the included studies had limitations, and there was a certain risk of bias. For example, the implementation of allocation concealment was not mentioned in the included studies, and it increased the possibility of human factors intervention and caused selection bias. Therefore, the interpretation of the results of this study should be cautious, and more clinical trials are needed to draw definitive conclusions in the future.

## Conclusion

According to this study, acarbose (50 or 100 mg each time, once daily at breakfast time) may attenuate the decrease of blood pressure after breakfast and reduce the occurrence of PPH in the treatment of patients with PPH and glucose metabolism disorders. More clinical trials are needed to demonstrate this conclusion in the future.

## Data Availability Statement

The original contributions generated for the study are included in the article/Supplementary Material, further inquiries can be directed to the corresponding author/s.

## Author Contributions

FX directed and supervised the study. BW and JZ designed the protocol. BW, JZ, QZ, BL, RW, and YZ were responsible for data collation and statistical analysis. BW wrote the first draft. JZ reviewed and checked the manuscript. All authors read and approved the final manuscript.

## Conflict of Interest

The authors declare that the research was conducted in the absence of any commercial or financial relationships that could be construed as a potential conflict of interest.

## Abbreviations

PPH: postprandial hypotension
SBP: systolic blood pressure
IGT: impaired glucose tolerance
DM: diabetes mellitus
MD: weighted mean difference
SMD: standard mean difference
OR: odds ratio
DBP: diastolic blood pressure
MBP: mean arterial blood pressure.
