# Supplementary material for: Acarbose for Postprandial Hypotension With Glucose Metabolism Disorders: A Systematic Review and Meta-Analysis
Source: Front Cardiovasc Med. 2021 May 20;8:663635. doi: 10.3389/fcvm.2021.663635 (PMC8172613; doi:10.3389/fcvm.2021.663635)
Supplement: Supplementary file 1 [file Table_1.DOCX]

Supplementary Material

**Supplementary Table 1  Search strategy for the Embase database**

| **Search Query** | |
| --- | --- |
| #11. | #7 AND #10 |
| #10. | #8 OR #9 |
| #9. | #7 AND #8 |
| #8. | glumida:ti,ab,kw OR glucobay:ti,ab,kw OR glucoba:ti,ab,kw OR 'bay g 5421':  ti,ab,kw OR prandase:ti,ab,kw OR precose:ti,ab,kw OR acarbose:ti,ab,kw OR 'acarbose'/exp |
| #7. | #5 OR #6 |
| #6. | 'postprandial hypotension'/exp OR 'postprandial hypotension':ti,ab,kw |
| #5. | #3 AND #4 |
| #4. | #1 OR #2 |
| #3. | 'postcibal periods':ti,ab,kw OR 'periods,postcibal':ti,ab,kw OR 'period,postcibal':ti,ab,kw OR 'postcibal period':ti,ab,kw OR 'postprandial periods':ti,ab,kw OR 'periods, postprandial':ti,ab,kw  OR 'period,postprandial':ti,ab,kw OR 'postprandial state':ti,ab,kw OR 'postprandial':ti,ab,kw |
| #2. | 'hypotension':ti,ab,kw OR 'low blood pressure':ti,ab,kw OR 'blood pressure, low':ti,ab,kw |
| #1. | 'hypotension'/exp |
