# Supplementary material for: Acarbose for Postprandial Hypotension With Glucose Metabolism Disorders: A Systematic Review and Meta-Analysis
Source: Front Cardiovasc Med. 2021 May 20;8:663635. doi: 10.3389/fcvm.2021.663635 (PMC8172613; doi:10.3389/fcvm.2021.663635)
Supplement: Supplementary file 2 [file Table_2.docx]

Supplementary Material

**Supplementary Table 2 Leave-1-out sensitivity analysis**

|  | **Incidence（95%CL）** | | |
| --- | --- | --- | --- |
| **Study omitted** | 1. **SBP** | 1. **DBP** | 1. **MBP** |
| Jie Zhang2014, et al | -11.08(-14.32, -7.84) | -5.28(-11.84, 1.28) | -9.16(-15.13, -3.18) |
| Jie Zhang2017, et al | -8.57(-11.80, -5.33) | -5.43(-12.17, 1.31) | -5.99(-8.92, -3.06) |
| Wei Qiao2016, et al | -10.37(-15.26, -5.48) | -9.26(-13.44, -5.07) | NA |
| Qian Peng2018, et al | -9.30(-14.06,-4.54) | -7.43(-16.50, 1.65) | -9.12(-15.83, -2.41) |

CI: confidence interval; SBP: systolic blood pressure; DBP: diastolic blood pressure; MBP: mean arterial pressure; NA: not applicable
